# A new earthworm species of the genus *Eisenia* Malm, 1877 (Oligochaeta, Lumbricidae) from the Ili River valley, northwestern China

**DOI:** 10.3897/zookeys.1285.192248

**Published:** 2026-07-16

**Authors:** Pingping Xu, Yufeng Zhang, Xin Shu, Dayong Han, Huifeng Zhao

**Affiliations:** 1 College of Biological Sciences and Technology, Yili Normal University, Yining 835000, Xinjiang, China Hebei Key Laboratory of Animal Diversity, College of Life Sciences, Langfang Normal University Langfang China; 2 Hebei Key Laboratory of Animal Diversity, College of Life Sciences, Langfang Normal University, Langfang 065000, China College of Biological Sciences and Technology, Yili Normal University Yining China

**Keywords:** Central Asia, DNA barcoding, mitochondrial genome, taxonomy

## Abstract

The present study focuses on the taxonomy of the earthworm genus *Eisenia* Ealm, 1877 in China. The taxonomic framework for this genus in China has long been ambiguous, with studies in the northwestern region being particularly scarce. Prior to this research, only four subspecies within the *Eisenia nordenskioldi* (Eisen, 1879) complex, distributed across northeastern and northern China, had been described. Based on detailed morphological characteristics and molecular data, this paper describes a new species, *E. ilensis***sp. nov**., collected from the Ili River valley in northwestern China. The key diagnostic characteristics of this new species include a medium body length (37–103 mm); two pairs of small, spherical spermathecae without ducts, located at the inter-segmental furrows 9/10 and 10/11; a pair of small, circular tubercula pubertatis on the clitellum situated between the ab setae; the first of dorsal pore beginning at the 4/5 inter-segmental furrow; and a pair of slit-like male pores on segment XV. Phylogenetic analyses based on the mitogenomic protein coding genes indicate that *E. ilensis***sp. nov**. forms a distinct clade with *E. balatonica* Pop, 1943 supporting its status as an independent species and a basal evolutionary status. The discovery of the new species of *E. ilensis***sp. nov**. not only enriches the species diversity of the genus *Eisenia* in China but also provides a crucial taxonomic foundation for subsequent research into the evolution and dispersal history of this genus in Eurasia.

## Introduction

Earthworms, as keystone macrofauna in terrestrial ecosystems (Darwin 1881; Phillips et al. 2019), function as vital “soil ecosystem engineers” that play a central role in regulating soil structure, nutrient cycling, and microbial dynamics (Jouquet et al. 2006; Kuzyakov and Blagodatskaya 2015; Xiao et al. 2021; Liu et al. 2022). Species within the family Lumbricidae Rafinesque-Schmaltz, 1815 often constitute a major portion of soil faunal biomass in Palearctic region (Csuzdi and Zicsi 2003). However, taxonomic studies on Lumbricidae remain challenging, partly due to the relatively simple anatomical structure of these invertebrates, which often makes accurate identification based solely on morphological evidence difficult (Bozorgi et al. 2019). Currently, Lumbricidae consists of 47 genera, 615 species and 74 subspecies, with its classification system undergoing continuous revision (Misirlioğlu et al. 2023). Phylogenetically considered Lumbricidae is one of the youngest families within the subclass Oligochaeta (Anderson et al. 2017; Erséus et al. 2020), it is believed to have originated in the Palearctic realm and is now widely distributed across all continents except Antarctica (Misirlioğlu et al. 2023).

Significant research progress has been made on the genus *Eisenia* Malm, 1877 in East Asia. For instance, Blakemore and Park (2012) described two new species, *E. gaga* Blakemore, 2012 and *E. sindo* Blakemore, 2012, from the Korean archipelago; Kawai et al. (2020) reported a new species, *E. nipponica* Kawai, 2020 from Japan. Hong and Csuzdi (2016) re-described *E. koreana* Zicsi, 1972 from North Korea, supplementing morphological and molecular data, and through DNA barcoding suggested that *E. nordenskioldi* populations in South Korea might contain unnamed independent lineages. Furthermore, phylogenomic studies by Shekhovtsov et al. (2017) revealed significant polyploidization and genotypic differentiation within this species in the Russian Far East. Recently, based on systematic sampling from multiple sites in northeastern China, Qin et al. (2025) established three new subspecies within the *E. nordenskioldi* complex: *E. n. shenzi* Qin, 2025, *E. n. jilinensis* Qin, 2025 and *E. n. chinensis* Qin, 2025, while significant genetic differentiation within this complex was also detected in northeastern China.

In contrast to other regions of China, taxonomic research on earthworms in northwest China, particularly in Xinjiang Uygur Autonomous Region, has been relatively limited. Although two families, six genera, and eight earthworm species have been recorded in Xinjiang (Xu and Xiao 2011), the documented diversity of the genus *Eisenia* remains notably scarce. It is widely accepted that the modern lumbricid fauna consists of components from different historical periods and origins, including Tertiary relict populations that have survived since the Eocene or Paleocene by adapting to specific environments (Omodeo 1952, 1956, 1961, 1988; Bouché 1972, 1983; Mršić and Šapkarev 1988; Mršić 1991; Misirlioğlu 2017; Marchán et al. 2020, 2022). The Ili River valley in Xinjiang Uygur Autonomous Region, located in the heart of Asia, with its unique topography and temperature inversion effect, mitigated the invasion of Siberian cold currents during the Quaternary glacial period, providing a refuge for Tertiary relict fauna (Chinese Academy of Sciences Xinjiang Comprehensive Survey Team, Institute of Botany, Chinese Academy of Sciences 1978). These stable and relatively isolated habitats allowed ancient earthworm lineages to persist through major geological and climatic events such as orogeny, acidification, and multiple glaciations.

Recently, an undescribed morpho-species of the genus *Eisenia* was discovered during a survey in the Ili River valley of Xinjiang. Therefore, this study aims to integrate morphological characteristics and molecular data to report and describe this new species, intending to provide supplementary information and updated data for the *Eisenia* earthworm fauna in China.

## Materials and methods

### Sampling

Earthworm specimens were collected during May 2025, in the Wild Walnut Nature Reserve (43.3496°N, 82.2732°E), Gongliu County, Yili Prefecture, Xinjiang Uygur Autonomous Region, China. Collection was performed by digging and hand sorting. Earthworm specimens were then preserved in 100% ethanol in the field and stored at -20 °C in the laboratory for subsequent morphological and molecular analyses.

### Morphological examination

External and internal characteristics were examined using a stereomicroscope (ZEISS) and ZEN 3.3 pro software for image capture to aid in the identification and measurement of morphological characteristics. Body length and width were measured; the external features including body coloration, number of segments, position of the prostomium, segment of the first dorsal pore, distance between male pores, number of segments covered by the clitellum and tubercula pubertatis, as well as the setal spacing at various segment positions were documented; the internal characters such as the spermathecae, crop, gizzard, calciferous glands, and seminal vesicles were examined. Generic identification and taxonomic assignment followed the classification system of Blakemore (2013).

### DNA extraction, amplification, and sequencing

Total genomic DNA was extracted from tail tissues of specimens using the TIANGEN Genomic DNA Kit (Beijing, China) following the manufacturer’s instructions. A 5’ fragment of the mitochondrial gene cytochrome c oxidase subunit I (COI) was amplified using the polymerase chain reaction (PCR). The PCR mixture (total volume 25 μl) contained 1 μl of template DNA, 17.25 μl of sterile dd H_2_O, 2.0 μl of dNTPs, 2.5 μl of Easy Taq Buffer, 0.25 μl of Easy Taq Polymerase, 1.0 μl of the forward primer LCO1490 (5'-GGTCAACAAATCATAAAGATATTGG-3') and the reverse primer HCO2198 (5'-TAAACTTCTGGGTGTCCAAAAAATCA-3') (Folmer et al. 1994). The PCR protocol was as follows: initial denaturation for 5 min at 95 °C; 35 cycles of denaturation for 30 sec at 95 °C, annealing for 30 sec at 51 °C, and extension for 45 sec at 72 °C; and a final extension for 5 min at 72 °C.

The PCR products were examined by electrophoresis in a 1% agarose gel and sent to Tianyi Huiyuan Biotechnology Co., Ltd. (Beijing, China) for sequencing using Sanger sequencing with an ABI 3730 automated sequencer. Sequences of FASTA format were aligned and edited using MEGA 5 software (Tamura et al. 2011).

In order to obtain the mitogenome of the new species, the genomic DNA of specimen-voucher CNWN17_01 and CNWN002 were sent to Novogene Science and Technology Co., Ltd. (Beijing, China) to perform the Next Generation Sequencing. The DNA was fragmented by ultrasonication, and a library with a fragment size of 250–300 bp was constructed for paired-end 150 base pairs (bp) library. Approximately two gigabytes of clean data of each voucher were generated. The mitogenomes were assembled using MitoZ v. 2.4 (Meng et al. 2019) and manually verified by comparison with the published mitogenomes of *Eisenia* available online. All sequences, including the COI gene and the mitogenomes, have been deposited in GenBank, with the accession numbers listed in Table 1.

**Table 1.** Specimens information provided and available online DNA data analyzed in this study.

**Specimen ID**	**Species**	**Genetic marker**	**Accession number**	**Reference**
CNWN002	*E. ilensis* sp. nov.	COI	PX552877	This study
CNWN17_01	*E. ilensis* sp. nov.	COI	PX552878	This study
CNWN17_02	*E. ilensis* sp. nov.	COI	PX552879	This study
CNWN17_04	*E. ilensis* sp. nov.	COI	PX552880	This study
H_wo63	*E. n. mongol*	COI	H_wo63	Blakemore 2013
H_wo65	*E. n. onon*	COI	H_wo65	Blakemore 2013
JX531477	*E. n. nordenskioldi* L2	COI	JX531477	Shekhovtsov et al. 2013
JX531479	*E. n. nordenskioldi* L2	COI	JX531479	Shekhovtsov et al. 2013
JX531489	*E. n. nordenskioldi* L3	COI	JX531489	Shekhovtsov et al. 2013
JX531491	*E. n. nordenskioldi* L3	COI	JX531491	Shekhovtsov et al. 2013
JX531500	*E. n. nordenskioldi* L5	COI	JX531500	Shekhovtsov et al. 2013
JX531501	*E. n. nordenskioldi* L5	COI	JX531501	Shekhovtsov et al. 2013
JX531522	*E. nordenskioldi pallida*	COI	JX531522	Shekhovtsov et al. 2013
JX531523	*E. nordenskioldi pallida*	COI	JX531523	Shekhovtsov et al. 2013
JX531526	*E. n. nordenskioldi* L4	COI	JX531526	Shekhovtsov et al. 2013
JX531527	*E. n. nordenskioldi* L4	COI	JX531527	Shekhovtsov et al. 2013
JX531538	*E. n. nordenskioldi* L1	COI	JX531538	Shekhovtsov et al. 2013
JX531539	*E. n. nordenskioldi* L1	COI	JX531539	Shekhovtsov et al. 2013
JX531571	*E. fetida*	COI	JX531571	Shekhovtsov et al. 2013
MK642870	*E. spelaea*	COI	MK642870	Shekhovtsov et al. 2020
MK642872	*E. balatonica*	COI	MK642872	Shekhovtsov et al. 2020
MK642871	*E. tracta*	COI	MK642871	Shekhovtsov et al. 2020
PQ059655	*E. n. chinensis*	COI	PQ059655	Qin et al. 2025
PQ059656	*E. n. chinensis*	COI	PQ059656	Qin et al. 2025
PQ059664	*E. n. shenzi*	COI	PQ059664	Qin et al. 2025
PQ059665	*E. n. shenzi*	COI	PQ059665	Qin et al. 2025
PQ059670	*E. n. jilinensis*	COI	PQ059670	Qin et al. 2025
PQ059671	*E. n. jilinensis*	COI	PQ059671	Qin et al. 2025
CNWN17_01	*E. ilensis* sp. nov.	mitogenome	PZ129109	This study
CNWN002	*E. ilensis* sp. nov.	mitogenome	PZ131132	This study
MK618510	*E. n. nordenskioldi*	mitogenome	MK618510	Shekhovtsov et al. 2020
MK618513	*E. n. nordenskioldi*	mitogenome	MK618513	Shekhovtsov et al. 2020
MK642868	*E. n. nordenskioldi*	mitogenome	MK642868	Shekhovtsov et al. 2020
MK642867	*E. n. nordenskioldi*	mitogenome	MK642867	Shekhovtsov et al. 2020
MK618512	*E. n. pallida*	mitogenome	MK618512	Zhao et al. 2022
OM213999	*E. n. pallida*	mitogenome	OM213999	Csuzdi et al. 2022
OM687887	*E. nordenskioldi*	mitogenome	OM687887	Zhao et al. 2022
OL840314	*E. nordenskioldi*	mitogenome	OL840314	Zhao et al. 2022
OK513069	*E. andrei*	mitogenome	OK513069	Csuzdi et al. 2022
OK513070	*E. fetida*	mitogenome	OK513070	Csuzdi et al. 2022
MK642870	*E. spelaea*	mitogenome	MK642870	Shekhovtsov et al. 2020
MK642871	*E. tracta*	mitogenome	MK642871	Shekhovtsov et al. 2020
MK618511	*E. nana*	mitogenome	MK618511	Shekhovtsov et al. 2020
MK642872	*E. balatonica*	mitogenome	MK642872	Shekhovtsov et al. 2020
MZ857199	*Bimastos parvus*	mitogenome	MZ857199	Liu et al. 2021

### Molecular species delimitation analyses

In this study, molecular species delimitation was performed using the COI data through the Assemble Species by Automatic Partitioning (ASAP) (Puillandre et al. 2021) and Generalized Mixed Yule Coalescent (GMYC) (Pons et al. 2006) methods. ASAP is an automated species delimitation approach based on the COI marker, which identifies genetic distance thresholds (i.e., barcode gaps) to determine whether individuals belong to the same species. The analysis was conducted on the online web server (https://bioinfo.mnhn.fr/abi/public/asap/) under the Kimura 2-parameter (K2P) (Kimura 1980) model. GMYC is a likelihood-based method that delimits species by fitting within- and between-species branching models. A bifurcating, rooted, ultrametric tree inferred using the BEAST package v. 1.7.5 (Drummond et al. 2012), with all parameters configured in BEAUTi v. 1.7.5, with single-locus data as input files, was submitted to GMYC analysis implemented in R 3.6.2 (R Core Team 2016) using the splits package v. 1.0–19 (Ezard et al. 2013). Additionally, pairwise genetic distances were calculated for genetically highly divergent groups using the K2P model and be performed in MEGA 5, and the results are presented in Table 2.

**Table 2.** Morphological comparison of similar species within the Genus *Eisenia*. Hyphen indicates data not available.

**Character**	***E. nana***	***E. spelaea***	***E. tracta***	***E. balatonica***	***E. nordenskioldi***	***E. ilensis* sp. nov**.	***E. n. nordenskioldi***	***E. n. shenzi***	***E. n. chinensis***	***E. n. polypapillata***
Length (mm)	-	55–110	-	40–100	-	43–67	25–44	38–81	30–50	65–70
Width (mm)	2.5–3	3–6	5–12	1.5–2.5	3.57–6.5	5–8.06	-	5.32–6.5	3.9–5.1	4–5
Segment	-	80–120	-	80–132	-	113–144	106–125	78–156	92–127	102–137
Color	-	yellowish-violet with paler ventral side	-	brown or yellow is brown	unpigmented to pale to different shades of purple	pale yellow with hints of brown	dark puce to pale	dorsal side dark-purple except the gray clitellum	pale	pale
1^st^ dorsal pore	-	4/5	-	4/5	4/5	4/5	-	4/5	4/5	4/5
Clitellum	xxvii,1/2 xxvii–xxviii, xxix–xxxii	(xxiv) xxv, xxvi, xxvii–xxxiii, xxxiv	xxvi–xxxiii	xxiv, xxv–xxx	xxiv–xxxiv	(xxiv) xxv–xxxi (xxxii)	xxvi, xxvii–xxxi	xxv, xxvi–xxxiii	1/2xxvi, xxvii–xxxii or xxvi, xxvii–xxxiii	xxvi, xxvii–xxxii
Tubercles	xxix-xxx	xxviii, xxix–xxxi, xxxii	xxix–1/2 xxxii	xxvi-xxix	xxvii–xxxii	xxvii–xxix	xxvii–xxxi	1/2xxviii, xxix-xxxi	xxviii, xxix-xxxi or 1/2xxviii, xxix-1/2xxxii	1/2xxviii, xxix-1/2xxxi,xxxi
Calciferous glands	-	xi–xiii without diverticula	-	xi, xii with diverticula	viii-xiii	x–xii without diverticula	-	xi–xii	xi–xii	-
Setal arrangement aa:ab:bc:cd:dd	-	5.75:1:4:1:15	-	4:1:4:1:5	-	1.99:0.46:2.10:0.30	-	1.95:0.34:1.54:0.27	1.48:0.22:1.13:0.21	-

### Phylogenetic analysis

Phylogenetic analysis was performed based on the 13 protein-coding genes (PCGs) of the mitogenomes of *Eisenia*, with *Bimastos parvus* (Eisen, 1874) selected as the outgroup. The PCGs were extracted from GenBank-format files using the script gbseqextractor.py (Meng et al. 2019), aligned with MAFFT (Katoh et al. 2002), and concatenated using the Perl script FASconCAT-G_v. 1.04.pl (Kück and Longo 2014).

Phylogenetic trees were reconstructed using both Maximum Likelihood (ML) and Bayesian Inference (BI) approaches. The ML analysis was conducted in RAxML 8.0 (Stamatakis 2014) under the GTRGAMMA model, employing the default rapid hill-climbing algorithm to search for the best-scoring tree. Nodal support was evaluated with 1,000 rapid bootstrap replicates. For BI analysis, the optimal nucleotide-substitution model (GTR + I + G) was selected with jModelTest 2.1 (Posada 2008; Darriba et al. 2012) according to the Akaike Information Criterion (Akaike 2003). The NEXUS-formatted alignment was analyzed in MrBayes v. 3.2.6 (Ronquist et al. 2012), running two independent Markov chain Monte Carlo simulations for two million generations, until the average standard deviation of split frequencies fell below 0.01. Resulting phylogenetic trees were visualized and edited in FigTree v. 1.4.4 (https://tree.bio.ed.ac.uk/software/figtree/).

## Results

### Morphological characterization

**Family Lumbricidae Rafinesque-Schmaltz, 1815**

**Genus *Eisenia* Malm, 1877**

#### Eisenia ilensis

Taxon classification

Animalia

LaminarialesLessoniaceae

Xu, Zhang & Zhao sp. nov.

77377654-CE68-54D4-B364-EC703037587B

https://zoobank.org/8D0A38CA-A929-443B-BE66-DDDA116096C7

Fig. 1

##### Material examined.

***Holotype*** • one clitellate (CNWN17_01, COI accession number: PX552878), Wild Walnut Nature Reserve (43.3496°N, 82.2732°E, 1221.64 m elev.), Gongliu County, Ili Prefecture, Xinjiang Uygur Autonomous Region, 2025-05-28, collected by Yufeng Zhang. ***Paratypes***: • three clitellates (CNWN 17_02, COI accession number: PX552878; CNWN17_04, COI accession number: PX552879; CNWN002, COI accession number: PX552877), same data as of the holotype.

**Figure 1.**
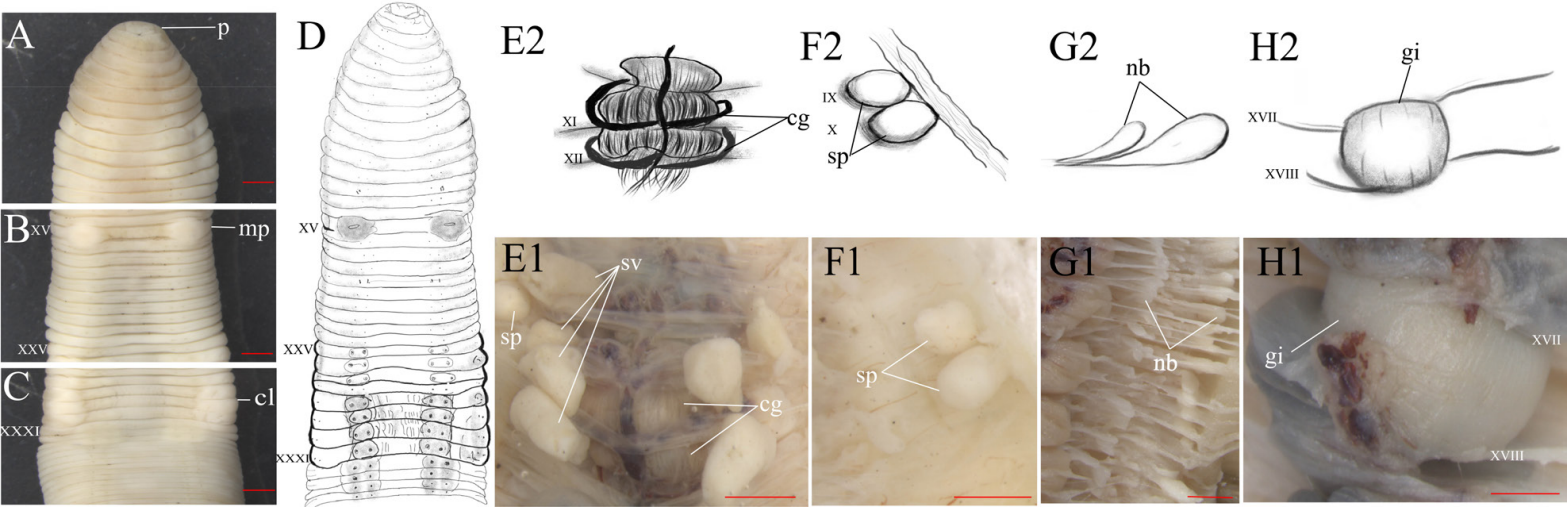
*Eisenia ilensis* sp. nov., holotype (CNWN17_01). **A**. Ventral view of the prostomium; **B**. Ventral view of the male pore; **C**. Ventral view of the clitellum; **D**. Overall ventral view; **E1**. Left spermathecae, seminal vesicles and calciferous glands; **E2**. calciferous glands; **F1, F2**. Right spermathecae; **G1, G2**. Right nephridial bladders; **H1, H2**. Gizzard. Scale bars: 1 mm. Abbreviations: cg, calciferous glands; cl, clitellum; gi, gizzard mp, male pore; nb, nephridial bladder; p, prostomium; sp, spermathecae; sv, seminal vesicles.

##### Additional material.

10 clitellates, same data as of the holotype. All the specimens of the new species have been deposited in Langfang Normal University, Hebei, China (C-HLU).

##### Diagnosis.

Body primarily pale yellow with hints of brown, length 43.0–67.0 mm, diameter 5.0–8.0 mm, number of segments 113–144. Prostomium open epilobic. Setae: lumbricine, four pairs, existing in each segment. First dorsal pores occur at 4/5. Two pairs of spermathecal pores in 9/10/11 close to mid-D. Female pore invisible and male pores in XV lateral. Clitellum within XXIV/XXV–XXXI, tubercula pubertatis within XXVII–XXIX, small cake-shaped. Four pairs of seminal vesicles in IX–XII. Spermathecae two pairs, ivory-white present on 9/10 and 10/11, the shape is spherical near the dorsal midline, without duct. Calciferous gland in segment X–XII, with one of gizzard in XVII–XVIII.

##### Description.

Body stout but relatively small. Pigmentation in alcohol pale yellow with a paler ventrum; clitellum white. Length 43.0–67.0 mm. Diameter 5.0–8.06 mm. Segments 113–144. Prostomium open epilobic. Dorsal pores from 4/5. Setae closely paired, four pairs per segment, appearing as small dots; setal ratio approximately aa = bc, ab = 1.5 cd. Genital markings present laterally on VIII, IX, and on the right side of XI, band-shaped. Tubercula pubertatis on XXVII, XXVIII, XXIX of the clitellum, small cake-shaped. The clitellum is saddle-shaped, spanning segments XXV–XXXI (slightly encroaching onto segment XXIV anteriorly or extending to segment XXXII posteriorly in some paratypes). Nephropores, spermathecal pores, and female pores invisible. Male pores relatively large, in segment XV lateral of setal line b; distance between male pores 3.23 mm (*n* = 6).

Internally, septum 12/13 thickened. Spermathecae two pairs, white and spherical; spermathecal duct absent, located in segments IX and X, positioned near the dorsal midline. Testes and funnels invisible. Seminal vesicles four pairs in segments IX–XII (smallest in IX and X, largest in XII). Ovaries invisible. Hearts in segments VI–IX. Calciferous glands in segments X and XII, fleshy, pink. Crop in segments XV–XVI. Muscular gizzard in segments XVII–XVIII. Excretory system holonephric, with digitate nephridia. Extra-esophageal vessels, testis sacs, epididymis, and typhlosole absent.

##### Distribution.

Ili Kazakh Autonomous Prefecture (Xinjiang Uygur Autonomous Region, China).

##### Habitat.

Litter or soil geophage (epigeic or endogeic ecological groups) inhabiting broad-leaved mixed forests and deciduous broad-leaved forests.

##### Etymology.

The specific name of the species refers to its distribution area in Ili, in the northwest of Xinjiang, China.

##### Remarks.

*Eisenia* is a Palearctic genus comprising approximately 50 valid taxa (e.g., *E. sibirica* Perel & Graphodatsky, 1984, *E. lagodechiensis* Michaelsen, 1910, *E. nana* Perel, 1985, etc.) (Hong and Csuzdi 2016). Common characteristics within the genus include dorsal pores commencing at the inter-segmental furrow 4/5 (although the first dorsal pore may occasionally occur at 3/4 or 5/6); the clitellum is predominantly positioned in segments XXVI–XXXII, with minor anterior or posterior extensions in some species; and male pores located in segment XV.

The new species is morphologically similar to *E. nordenskioldi* and *E. balatonica* (Table 2), which occur in the Siberian region (Eisen 1879; Pop 1943). They are similar in the number and position of clitellar segments, the position of calciferous glands, the arrangement of setae, the location of dorsal pores, and body diameter. However, *E. ilensis* sp. nov. is distinguished from *E. nordenskioldi* and *E. balatonica* by the following characteristics. Body coloration: the new species is pale yellow (in alcohol-preserved specimens) with a paler ventrum, and the clitellum is pure white; whereas *E. nordenskioldi* exhibits a dark purplish-brown coloration, and *E. balatonica* is dark red-brown. Spermathecal structure: the spermathecae duct of the new species absent, while both *E. nordenskioldi* and *E. balatonica* possess a distinct duct. In *E. ilensis* sp. nov., the tubercula pubertatis of the clitellum are located in segments XXVII–XXIX, whereas in *E. nordenskioldi* they are shifted posteriorly to segments XXIX–XXXI. Additionally, the genital markings in *E. ilensis* sp. nov. are positioned more anteriorly (segments VIII–XI), compared to those in *E. nordenskioldi*, which are located further posteriorly (segments XXVII–XXXI).

In addition, the new species exhibits distinct morphological differences from other subspecies within the *E. nordenskioldi* complex. In terms of body size, the new species is similar to *E. n. shenzi* but significantly larger than *E. n. chinensis* and smaller than *E. n. polypapillata* Perel, 1969. These four taxa can be clearly distinguished by their body coloration: the new species is pale yellow; *E. n. shenzi* is grayish-purple to pinkish-brown; *E. polypapillata* is deeply pigmented, with a dark purple to dusky brown dorsum; and *E. n. chinensis* is pale red to pinkish-gray. Regarding body width, the new species (5.0–8.06 mm) is among the more robust forms in the comparison (*E. n. chinensis*, *E. n. polypapillata*). In the arrangement of genital markings, the new species exhibits band-like lateral papillae distributed on the right side of segments VIII, IX, and XI. In contrast, the papillae of *E. n. shenzi* occur as paired small round spots along the ab line in segments XXVI–XXXI; those of *E. n. polypapillata* and *E. n. chinensis* are round spots confined to the cd line in segments IX–XII. The new species possesses small cake-shaped tubercula pubertatis and a gizzard occupying segments XVI–XVIII (spanning three segments). By comparison, the tubercula pubertatis of the other three subspecies are not described as cake-shaped, and their gizzards all begin in segment XVII, covering only two segments.

### Molecular species delimitation

**Figure 2.**
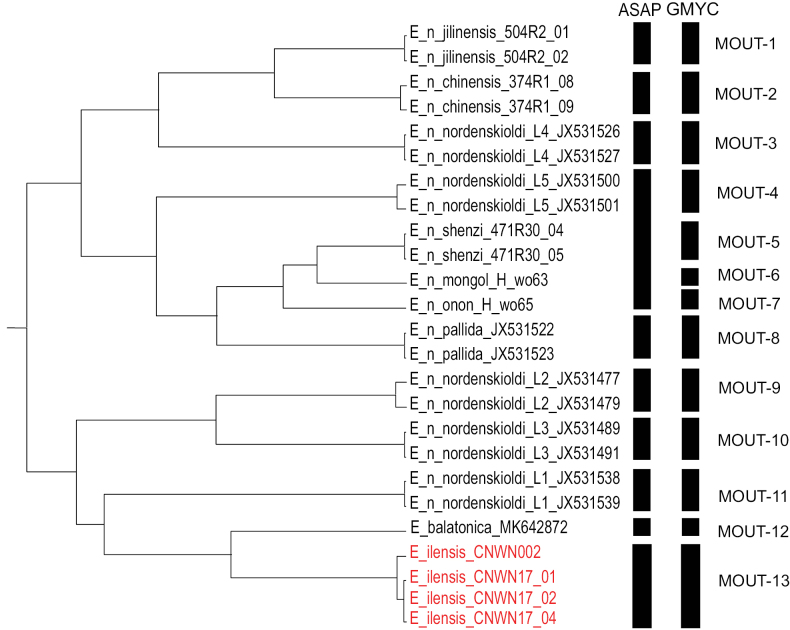
Molecular species delimitation of the *E. ilensis* sp. nov. and similar morph-species in *Eisenia*. ASAP, automated barcode gap discovery; GMYC, Generalized Mixed Yule Coalescent model. Red tips indicate the new species.

Based on 25 COI sequences from the novel species and its close relatives, the GMYC analysis integrating the Yule model and coalescent theory delineated the COI sequences into 13 Molecular Operational Taxonomic Units (MOTUs). Specifically, MOTU-1 consists of *E. n. jilinensis*; MOTU-2 consists of *E. n. chinensis*; MOTU-3 consists of *E. n. nordenskioldi* L4; MOTU-4 consists of *E. n. nordenskioldi* L5; MOTU-5 consists of *E. n. shenzi*; MOTU-6 consists of *E. n. mongol*; MOTU-7 consists of *E. n. onon*; MOTU-8 consists of *E. n. pallida*; MOTU-9 consists of *E. n. nordenskioldi* L2; MOTU-10 consists of *E. n. nordenskioldi* L3; MOTU-11 consists of *E. n. nordenskioldi* L1; MOTU-12 consists of *E. balatonica*; and MOTU-13 consists of the novel species *E. ilensis* (Fig. 2). In contrast, the ASAP divided the COI sequences into 10 MOTUs. The primary difference compared to the GMYC results is that ASAP grouped *E. n. nordenskioldi* L5 and *E. n. shenzi* with the two Mongolian subspecies (*E. n. mongol* and *E. n. onon*) into a single MOTU.

The K2P analysis of COI sequences, the genetic divergence between *E. ilensis* sp. nov. and its congeners ranged from 15% to 28% (Table 3), exceeding the commonly used species delimitation threshold of 15% for earthworms (Han et al. 2024, 2026; Liu et al. 2025a, 2025b, 2026; Zhao et al. 2025; Qiao et al. 2026). Within the *E. nordenskioldi* species complex, the divergence among different subspecies ranged from 0 to 23.8%.

**Table 3.** Percentage of K2P distances of COI of *E. ilensis* sp. nov. and the other species/lineages of the genus *Eisenia* (values in %).

	**Lineage**	**1**	**2**	**3**	**4**	**5**	**6**	**7**	**8**	**9**	**10**	**11**	**12**	**13**	**14**	**15**
**1**	*E. ilensis* sp. nov.															
**2**	*E. n. pallida*	23.9														
**3**	*E. n. onon*	22.7	16.2													
**4**	*E. n. shenzi*	24.1	17.1	7.4												
**5**	*E. n. mongol*	23.9	18	8.2	8.4											
**6**	*E. n. nordenskioldi* L2	21.7–22.4	19.8–20.5	8.2–21.6	14.0–14.9	12.7–13.5										
**7**	*E. n. nordenskioldi* L5	22.2–23.4	20.3–21.3	16.0–17.4	16.5	14.4–15.0	15.5–18.2									
**8**	*E. n. nordenskioldi* L4	21.7–22.4	21.0–21.5	20.9–21.2	19.5–19.7	20.9–21.1	19.5–21.5	20.4–22.8								
**9**	*E. n. jilinensis*	19.3–19.6	18.8–19.0	16.5–16.9	19.3–19.5	16.9–17.4	16.7–17.8	20.1–21.1	16.9–17.8							
**10**	*E. n. chinensis*	21.0–21.4	19.5–20.0	17.6–18.1	17.9–18.6	19.4–20.2	17.1–18.8	19.9–20.4	17.1–18.0	9.2–10.0						
**11**	*E. n. nordenskioldi* L3	21.9–22.9	18.3–22.8	18.8–22.4	19.0–21.9	21.4–22.8	18.1–22.1	18.8–21.9	19.8–23.4	18.1–21.0	16.9–20.4					
**12**	*E. n. nordenskioldi* L1	19.6–22.2	20.0–23.8	19.0–20.9	14.0–21.3	14.6–21.6	0.0–21.6	16.1–22.5	19.7–21.6	16.9–17.6	18.3–21.3	17.8–22.8				
**13**	*E. balatonica*	15	23.8	22.9	22	22.6	20.8–21.5	21.0–23.2	21.9–22.1	20.0	22.2–23.0	19.3–21.7	18.0–21.3			
**14**	*E. tracta*	23.4	20.6	17.7	17.6	18.2	19.5–21.2	20.2–21.8	20.2–20.5	18.3–19.0	17.9–18.3	19.0–21.2	19.4–21.2	23.0		
**15**	*E. fetida*	24.4	22.5	24.3	24.7	17.4	22.7–23.2	21.1–23.1	23.2–23.4	21.4–22.2	22.3–22.6	23.1–25.1	23.2–26.4	26.5	21.6	
**16**	*E. spelaea*	28	28.2	23.6	23.9	23.6	24.7–25.5	25.1–25.8	25.0–25.2	24.8–25.0	24.4–24.7	26.0–28.1	23.7–25.5	28.0	21.9	21.7

### Phylogenetic relationships of mitogenomes

**Figure 3.**
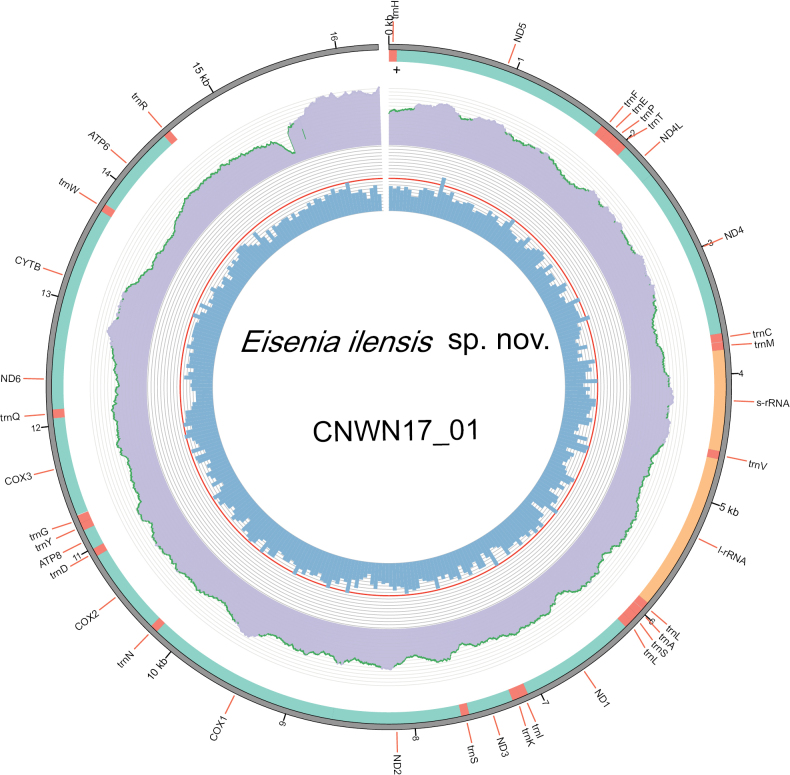
Mitogenome of *Eisenia ilensis* sp. nov. (Holotype of CNWN17_01, and that of the paratype of CNWN002 is same as the holotype and is not shown) The inner circles indicate the GC content in each 50-site window, and the coverage depth by purple; the outer circle shows the arrangement of the genes, cyan for the PCGs, salmon for tRNAs, orange for rRNAs.

**Figure 4.**
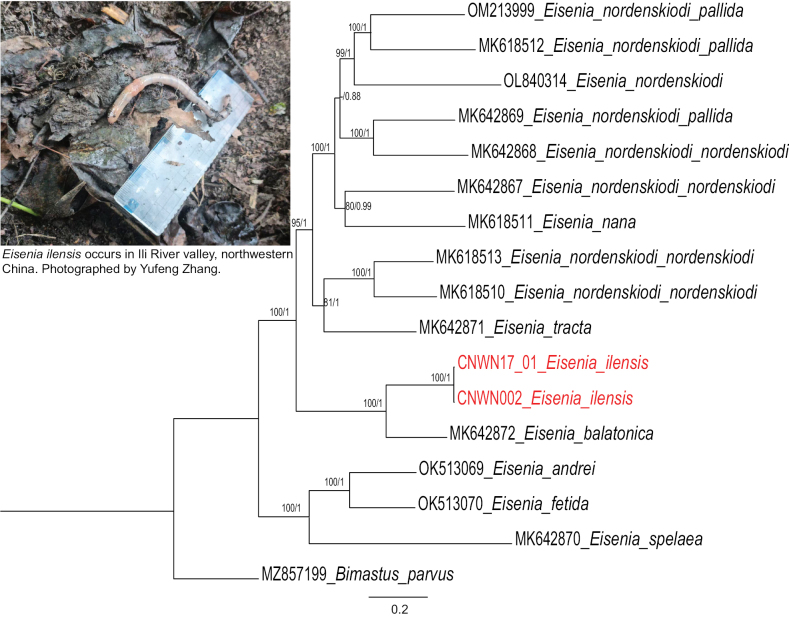
Phylogenetic tree of *E. ilensis* sp. nov. and other species in the *E. nordenskioldi* complex and outgroup taxa based on the 13 protein coding genes of the mitogenomes using the maximum likelihood method and Bayesian inference. Numbers near branches indicate the maximum likelihood bootstrap support/Bayesian posterior probabilities. Bootstrap values lower than 70 and posterior probability values lower than 0.80 indicate weak support and are not shown. Red tips indicate the new species. Top left figure shows an *Eisenia ilensis* sp. nov. alive specimen in Wild Walnut Nature Reserve, Gongliu County, Xinjiang, China.

The newly sequenced mitogenomes are shown in Fig. 3, and they contain 37 genes, comprising 13 protein-coding genes, two ribosomal RNA genes, 22 transfer RNA genes, and a putative control region. The phylogenetic trees constructed based on 13 protein-coding genes from mitogenomes available online using Maximum Likelihood (ML) and Bayesian Inference (BI) methods exhibit congruent topological structures (bootstrap support ≥ 50 or posterior probability ≥ 0.80) (Fig. 4). In the phylogenetic trees, *E. ilensis* sp. nov. forms an independent lineage with maximal statistical support (bootstrap value 100, posterior probability 1.00). Its branching relationships with other *Eisenia* species, such as *E. balatonica* and *E. nordenskioldi*, also receive the highest statistical support. Furthermore, *E. ilensis* sp. nov. and *E. balatonica* are recovered as sister taxa with full support (bootstrap value 100 and posterior probability 1.00). This sister group further clusters with a clade comprising multiple subspecies of *E. nordenskioldi* to form a highly supported monophyletic group, indicating a close sister-group relationship between the new species and these *E. nordenskioldi* subspecies at a higher taxonomic level. Simultaneously, the *E. nordenskioldi* complex is confirmed as a monophyletic clade, within which three independent subclades can be distinguished, each with high bootstrap support of 100/1.00. However, *E. n. pallida* is not monophyletic, as its lineages are distributed among different subclades of the nominate subspecies *E. n. nordenskioldi*.

## Discussion

The taxonomy of the genus *Eisenia* has long been controversial, primarily due to frequent conflicts between morphological and molecular evidence. Sole reliance on morphology can be problematic: current earthworm taxonomy depends almost entirely on a limited number of external and internal morphological characters, with reproductive system features being the most critical (Jamieson 2006). However, due to the relatively simple and stable life of earthworms in soil and the lack of specialized copulatory organs, they exhibit a phenomenon of “morphological stasis” (Novo et al. 2012; Pérez-Losada et al. 2012). This results in their phenotypic traits potentially retaining plesiomorphies, thereby obscuring evolutionary relationships. On the other hand, molecular studies (particularly those based on mitochondrial DNA) sometimes reveal unexpected phylogenetic positions, adding to the complexity. These challenges highlight the necessity of adopting an integrative approach, combining molecular data with morphological and ecological evidence to resolve taxonomic ambiguities. Hong and Csuzdi (2016) employed precisely this synthetic method to re-evaluate the unpigmented form of *E. nordenskioldi* on the Korean Peninsula, ultimately elevating it to a distinct species.

Against this background, our phylogenetic analyses strongly support the newly described species, *E. ilensis* sp. nov., as an independent lineage within the genus *Eisenia* (Fig. 4). Its basal position within the genus indicates an early divergence event, confirming it as a relatively ancient species. However, it should be noted that our phylogenetic reconstruction includes only a subset of *Eisenia* species, and thus our conclusions regarding the genus-level phylogeny remain preliminary. Notably, *E. ilensis* is not a derived branch of known East Asian groups; instead, it forms a highly supported sister clade (bootstrap value of 100 and posterior probability of 1.00) with the Siberian and European-distributed *E. balatonica*. This phylogenetic relationship strongly suggests that *E. ilensis* sp. nov. likely represents an ancient divergence event, with a lineage history independent of the recently radiated *E. nordenskioldi* complex in East Asia.

Morphological measurements of newly collected specimens revealed that relying solely on external morphological characters (such as body color, clitellum, etc.) cannot reliably distinguish it from *E. n. nordenskioldi*. The only morphological difference lies in the absence of a short duct at the spermathecal pores, a feature that can be used to differentiate it from *E. n. nordenskioldi*. Furthermore, the study found a lack of systematic morphological diagnostic data supporting the internal lineages of *E. n. nordenskioldi*. Although *E. fetida* (Savigny, 1826) and *E. andrei* Bouché, 1972 are extremely similar in morphological characters, they exhibit subtle differences. *Eisenia fetida* is a clitellate species, with intersegmental grooves lacking pigment, giving the body a pale or yellowish hue (hence named the “tiger worm” or “brandling worm”) (Pérez-Losada et al. 2005). In contrast, *E. andrei* is known as the “red worm,” possessing a uniform reddish body color pattern. This high similarity makes their morphological identification extremely difficult, often leading to misleading taxonomic literature. However, André (1963) initially considered these species as different morphs of *E. fetida* based on their pigmentation patterns. Subsequently, Bouché (1972) classified them as subspecies, namely *E. foetida fetida* and *E. foetida unicolor*.

This study employed the GMYC and ASAP methods for species delimitation. These methods were chosen because they can automatically execute computational algorithms, thereby minimizing human error in the results. Although the species boundaries obtained by different delimitation methods are largely consistent, they are not identical. GMYC divided the new species and its close relatives into 13 MOTUs, while ASAP divided them into 10. The main difference lies in ASAP grouping *shenzi*, the two Mongolian subspecies (*E. n. mongol* and *E. n. onon*), and *E. n. nordenskioldi* L5 into a single MOTU, whereas GMYC divided them. Carstens et al. (2013) suggested that such discrepancies may stem from differences in the methods’ ability to detect cryptic lineages or from violations of the underlying assumptions of the delimitation methods. They recommended caution in interpreting species delimitation results to avoid defining taxonomic units that do not accurately represent evolutionary lineages. Therefore, subsequent studies could adopt multi-locus data, such as mitogenome or nuclear markers, for species delimitations to address potential limitations of single-gene methods.

The newly described *E. ilensis* sp. nov. was discovered in the Wild Walnut Nature Reserve of the Ili River valley in Xinjiang, China. Its distribution results from the interaction between species-specific adaptations and the unique climate conditions of the Ili River valley. From a regional climatic perspective, the Ili River valley exhibits a west-opening, trumpet-shaped topography that captures moisture transported by the westerlies, thereby maintaining a locally humid environment amid the widespread aridification of inland Asia. The type location, Yehetao Gully, represents a typical ravine habitat within the valley, which benefits not only from this regional humidity but also a microclimate characterized by low winter temperatures due to the combined effects of its gully topography and relatively high elevation (800–1700 m). According to records from the Gongliu County Meteorological Bureau, the urban area (elevation 800 m) temperatures below –24 °C appeared in multiple days during December 2020 and January 2021, while temperatures in the higher-elevation Wild Walnut Nature Reserve are generally even lower (Liu et al. 2025c). Notably, species within *Eisenia* generally exhibit strong cold tolerance (Meshcheryakova and Berman 2014). For example, the adults of the *E. n. nordenskioldi* can withstand freezing temperatures as low as –35 °C, and their cocoons can endure extreme lows of –45 °C (Berman and Leirikh 1985). The subspecies *E. n. pallida* also demonstrates comparable cold resistance, with some individuals capable of reviving after exposure to temperatures between -30 °C and -35 °C (Meshcheryakova and Berman 2014). As endogeic earthworms, they can migrate to deeper, warmer soil layers to avoid extreme cold at the surface. Therefore, the inherent cold tolerance of the genus, combined with the specific microclimate—featuring both humidity and low winter temperatures—in the Ili River valley, together constitute key factors enabling the successful speciation of *E. ilensis* sp. nov.

## Conclusions

In the present investigation, an integrative methodology incorporating both morphological and molecular data was utilized to delineate a novel earthworm species, *E. ilensis* sp. nov., which was discovered in Northwest China. The identification of this new earthworm species serves to significantly enrich the documented biodiversity of the Ili River Valley. Moreover, it offers crucial insights for elucidating the structure and functioning of the local ecosystems. Additionally, it provides essential baseline data and constitutes a valuable case study for biodiversity conservation and monitoring initiatives. As the accumulation of survey data in the Ili River Valley region, it is anticipated that additional new species will be discovered and described in the future, further contributing to the biodiversity records of western China.

## Supplementary Material

XML Treatment for Eisenia ilensis

## Additional information

### Artificial Intelligence (AI) use

The authors accept full responsibility for the content of the manuscript, including the disclosure of any use of AI.

No AI tools were used in the preparation of this manuscript.

### Author ORCIDs

P. Xu https://orcid.org/0009-0009-1325-2251

Y. Zhang https://orcid.org/0000-0002-4066-6355

H. Zhao https://orcid.org/0000-0003-4243-9671

### Data availability

All of the data that support the findings of this study are available in the main text.
